# Effects of Elevated [CO_2_] and Low Soil Moisture on the Physiological Responses of Mountain Maple (*Acer spicatum* L.) Seedlings to Light

**DOI:** 10.1371/journal.pone.0076586

**Published:** 2013-10-17

**Authors:** Gabriel Danyagri, Qing-Lai Dang

**Affiliations:** Faculty of Natural Resources Management, Lakehead University, Thunder Bay, Ontario, Canada; University College Dublin, Ireland

## Abstract

Global climate change is expected to affect how plants respond to their physical and biological environments. In this study, we examined the effects of elevated CO_2_ ([CO_2_]) and low soil moisture on the physiological responses of mountain maple (*Acer spicatum* L.) seedlings to light availability. The seedlings were grown at ambient (392 µmol mol^−1^) and elevated (784 µmol mol^−1^) [CO_2_], low and high soil moisture (M) regimes, at high light (100%) and low light (30%) in the greenhouse for one growing season. We measured net photosynthesis (*A*), stomatal conductance (*g*_s_), instantaneous water use efficiency (IWUE), maximum rate of carboxylation (*V*_cmax_), rate of photosynthetic electron transport (*J*), triose phosphate utilization (TPU)), leaf respiration (*R*_d_), light compensation point (LCP) and mid-day shoot water potential (Ψ_x_). *A* and *g*_s_ did not show significant responses to light treatment in seedlings grown at low soil moisture treatment, but the high light significantly decreased the *C*_i_/*C*_a_ in those seedlings. IWUE was significantly higher in the elevated compared with the ambient [CO_2_], and the effect was greater at high than the low light treatment. LCP did not respond to the soil moisture treatments when seedlings were grown in high light under both [CO_2_]. The low soil moisture significantly reduced Ψ_x_ but had no significant effect on the responses of other physiological traits to light or [CO_2_]. These results suggest that as the atmospheric [CO_2_] rises, the physiological performance of mountain maple seedlings in high light environments may be enhanced, particularly when soil moisture conditions are favourable.

## Introduction

The responses of shade tolerant species to light availability in canopy gaps are at two endpoints. Some shade tolerant species grow slowly and consistently in the understory and do not respond considerably to canopy gaps. On the other hand, some persist in the understory and only show considerable increases in growth once canopy gaps are created [1], [2]. Additionally, some shade tolerant species exhibit light foraging growth habits that enable them to exploit canopy gaps [3]. Species responses to the occurrence of canopy gaps can be important in influencing forest dynamics. However, the responses of shade tolerant species to canopy gaps may be limited by physiological constraints such as decreased photosynthesis if other resources are limiting [4], [5]. Therefore, shade tolerant species responses to canopy gaps may be contingent on their ability to alter physiological traits for positive carbon balance [6]. While light availability in canopy gaps is considered a primary determinant of photosynthetic capacity [7], [8], other factors may influence net photosynthesis (*A*). Any factors that enhance the ability of shade tolerant species to increase *A* in canopy gaps may play an important role in forest dynamics.

The atmospheric [CO_2_] has been increasing since the industrial revolution, and carbon-climate models predict the increase to continue [9]. An increase in the atmosphere [CO_2_] alone has, aside from affecting global climate, instant effect on plants, and thus terrestrial carbon storage [10], [11]. Because CO_2_ is the primary substrate for photosynthesis, and the photosynthetic machinery of C_3_ plants is able to handle higher than the current [CO_2_], the increase in [CO_2_] is predicted to have a ‘fertilization’ effect on C_3_ plants [12]–[14]. The positive effect of elevated [CO_2_] on the photosynthetic rate is related to increased activity of the photosynthetic enzyme, ribulose-1, 5-bisphosphate carboxylase/oxygenase (Rubisco) and inhibition of photorespiration due to the shift in CO_2_: O_2_ ratio [15], [16]. Elevated [CO_2_] also has been shown to increase apparent quantum efficiency (AQE) and lower the light compensation point (LCP) of *A*, and thus increasing photosynthetic carbon gain [17]–[19]. Numerous studies have reported increases in photosynthesis under elevated [CO_2_], at least in the short term [20]–[22]. Higher absolute photosynthetic rates in high light environment [7], [23], suggest that elevated [CO_2_] can be expected to further increase photosynthetic carbon gain in high light environments. However, environmental stresses can influence the positive interaction of light and [CO_2_] on net photosynthesis. A reduction in soil volumetric water content reduced biomass and photosynthesis of well-lit *Pinus taeda* seedlings growing at cool and warm sites under ambient and elevated temperature and [CO_2_] [24]. A reduction in soil moisture substantially decreased the positive effect elevated [CO_2_] on *Pinus taeda* L. seedlings growing at the northern, central and southern sites in its native range [25]. Tschaplinski *et al*. [26] also reported that low soil moisture reduced the photosynthetic response of *Liquidambar styraciflua* L. and leaf area production of *Acer saccharum* Marsh. to elevated [CO_2_] under high light environments. However, the effect of [CO_2_] and soil moisture on the physiological responses of plants to high light regime is less well understood. A good understanding of the effects of climate change on shade tolerant species in simulated canopy gaps may be critical for a reliable prediction of forest succession in the future.

The changes in the global climate are predicted to be accompanied by a 1.4–5.8°C increase in global mean temperatures by the end of this century [9], [27], [28]. Increases in temperature will likely cause a decrease in soil moisture due to increased rate of evapotranspiration [29], [30]. The negative effect of low soil moisture on mature trees [31]–[33], seedlings and saplings [34]–[36] has been demonstrated. Plants that are growing at low soil moisture conditions have lower photosynthetic rates primarily because of decreased stomatal conductance [37]–[40]. Therefore, we hypothesize that low soil moisture would limit elevated CO_2_ stimulation of photosynthesis, and that low soil moisture causes a greater reduction of photosynthesis in seedlings grown in high than low light conditions.

In the present study, the physiological responses of mountain maple (*Acer spicatum* L.) seedlings to light under different [CO_2_] and soil moisture were evaluated. Mountain maple is an important understory shrub or tree species in the boreal mixedwood that contributes immensely to the composition, structure and diversity of such forests [41], [42]. It influences the amount of light reaching the forest floor, thus affecting the growth of other plant species [43]. Mountain maple grows on a wide range of habitats and persists through all the stages of forest development [44]. Its phenotypic plasticity in response to light allows it to acclimate to a large range of light conditions and responds rapidly to canopy gaps after removal of the overstory vegetation [3], [45]. Previous studies have also indicated that mountain maple is sensitive to low soil moisture conditions [46]. Despite the evidence that mountain maple responds rapidly to canopy gaps, there is still a lack of information on how low soil moisture may affect mountain maple’s physiological responses to light under future climate.

## Materials and Methods

### Plant Materials

The Jack Haggerty Forest, where the mountain maple seeds were collected, is the research site of Lakehead University. The study did not involve protected or endangered species. The Jack Haggerty Forest is located approximately 37 km north of Thunder Bay, ON (48°22′56″ N, 89°14′46″ W). Seeds were soaked in a 1000 µmol mol^−1^ giberellic acid (GA) for 24 hrs and thenkept at 4°C temperature for two months on moistened paper towels. The seed coats were cracked to facilitate germination after the stratification. Germination (November 02–09, 2011) occurred in a 2∶1 mixture of vermiculite and peat moss in horticultural trays. The average environmental conditions in the greenhouse during the germination were: 22/16°C day/night temperature and a photoperiod of 16 hr (maximum summertime photoperiod for Thunder Bay, ON, according to Environment Canada Weather Report, 2010). The growing medium was maintained moist by sprinkling water daily. Three weeks after germination was completed, a total of 160 relatively uniform-sized seedlings were transplanted into plastic containers (31.5 cm deep, 11 cm top diameter and 9.5 cm bottom diameter) with the same growing medium composition as described above.

### Experimental Design

The experiment had two [CO_2_], two light and two soil moisture treatments in a split-split-plot experimental design. The [CO_2_] (two replicates per treatment) were ambient (392 µmol mol^−1^) and elevated (784 µmol mol^−1^). Argus CO_2_ generators (Argus systems Ltd, Vancouver, BC, Canada) were used to achieve the CO_2_ elevation. The sub-plot treatment consisted of two levels of light (100% and 30% of full light in the greenhouse (averaged 650 µmol m^−2^s^−1^PAR at the seedling height). Neutral density shade cloth (supported on metal frames) was used to obtain the 30% light level (i.e., the shading reduced PAR by 70%). High pressure sodium lamps were used to provide supplemental light on cloudy days and to extend the photoperiod to 16 hr. The sub-sub-plot treatment comprised of two (high and low) soil moisture treatments within each sub-plot. In the high soil moisture (average water content of 0.28 cm^3^ cm^−3^) treatment, the seedlings were watered to the dripping point daily. The seedlings in the low soil moisture (average water content of 0.14 cm^3^ cm^−3^) treatment were watered every 2–4 days depending on soil moisture measurements. The watering was done when the moisture levels in low soil moisture treatment fall below 40% compared with the high soil moisture treatment. The moisture level of the growing medium was measured daily using a HH2 moisture meter (Delta-T Devices Ltd, Cambridge, UK). The low soil moisture treatment started one week after the seedlings were transplanted to allow for the establishment of root-growing medium contact.

The environmental conditions of the greenhouses were set at 22/16°C day/night air temperature and relative humidity of 50%. The environmental conditions were controlled and monitored by an Argus environmental control system (Argus, Vancouver, BC, Canada). All the seedlings were fertilized twice a week with a solution containing100, 15, 57, 6, 6 and 11 mg/L of N, P, K, Ca, Mg and S, respectively. The fertilization was done during the days when watering was needed. The fertilizer solution was formulated based on other studies on *Acer* species and other deciduous tree species [47].

### Photosynthetic Light and CO_2_ Responses

The light response curves of photosynthesis at the corresponding growth [CO_2_] were measured at seven PAR levels: 1100, 800, 400, 100, 60, 10 and 0 mol m^−2^ s^−1^ on a mature leaf (4^th^–6^th^ on the terminal shoot). The measurement was carried out on five randomly selected seedlings from each treatment combination. The measurement was done between 10∶00–15∶00 h with a LI-COR 6400 open gas exchange system (LI- 6400, LI-COR Biosciences, Lincoln, NE, USA). The relative humidity (RH) and temperature within the leaf chamber were set at 50% and 22°C, respectively. The LCP of photosynthesis, apparent quantum efficiency (AQE) and leaf respiration (*R*_d_) were calculated using the Photosyn Assistant software (Dundee Scientific, Scotland, UK).

The photosynthetic responses to [CO_2_] (*A*/*C*_i_ curves) were measured on the same seedlings and leaves used in the light response measurements. The measurements were taken at 50, 100, 200, 400, 800, 1000 and 1500 µmol mol^−1^ CO_2_ at 600 µmol m^−2^ s^−1^(saturating) PAR, 50% RH and 22°C leaf temperature. The net photosynthetic rate (*A)* and stomatal conductance (*g*_s_) were expressed on a leaf area basis. The response curves were analyzed using the Curve Fitting Utility 1.1 developed by Sharkey *et al.* [48] to estimate the maximum rate of Rubisco carboxylation (*V*_cmax_), photosynthetic electron transport rate (*J*), triose phosphate utilization (TPU) and dark respiration (*R*_d_). They were adjusted to values at 22°C leaf temperature [48] because actual leaf temperatures differed from 22°C as a result of different transpiration rates.

### Xylem Water Potential Measurements

The seedlings used in the above measurements were also used to measure midday xylem water potential (Ψ_x_). The measurement was done on the terminal shoot with a Scholander pressure chamber (PMS Instruments, Corvallis, OR) between 12∶00 and 15∶00 hours.

### Statistical Analysis

The data were analyzed with Data desk 6.01 Statistical Package (Data Description 1996). The assumptions of normality of distribution and homogeneity of variance were examined graphically using probability plots of the residuals and histograms, respectively. Since both assumptions were met, the analysis of variance (ANOVA) was done on the original data. The effects of [CO_2_], light, soil moisture, and their interactions were tested using a three-way ANOVA for split-split-plot design. The significant level was set at *P*≤0.05 but *P*-values ≤0.10 were considered marginally significant due to small number of replications and sample size in the study [49], [50]. Scheffé’s *post hoc* comparison of means was done when an interaction was significant.

## Results

**Figure 1:**
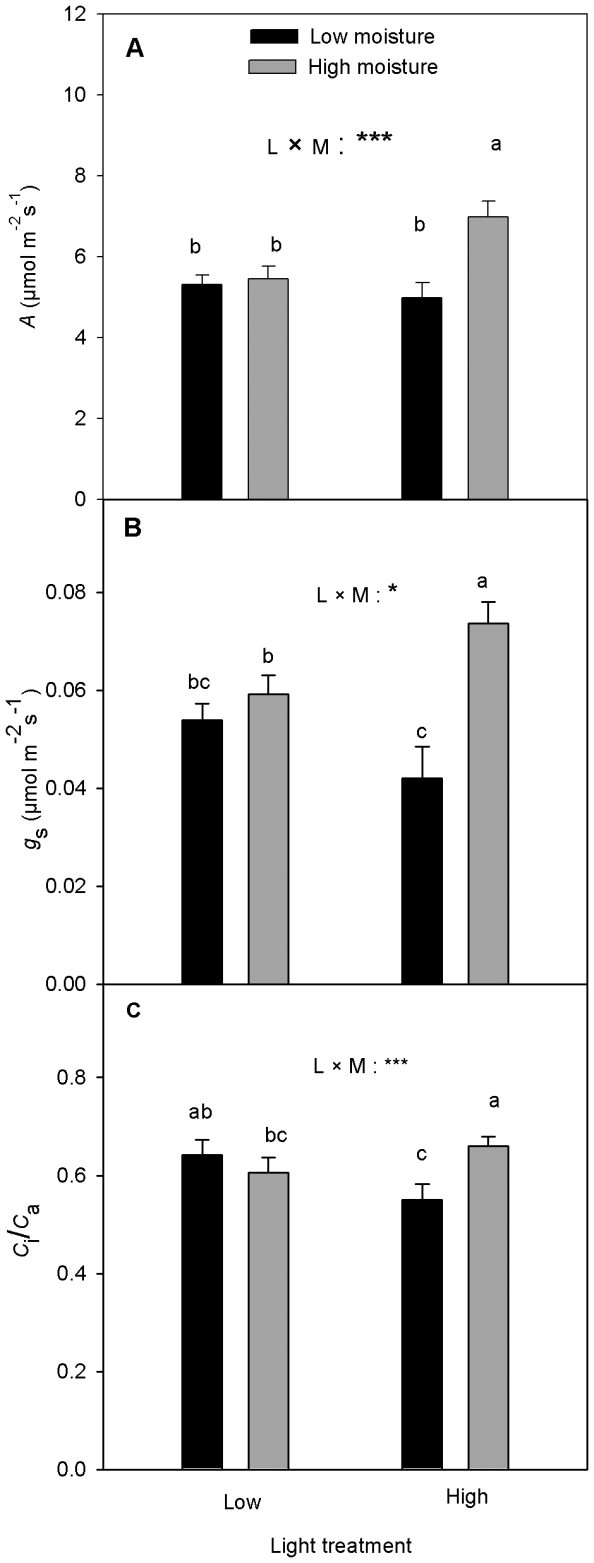
Effects of [CO_2_], light (L) and soil moisture (M) on net photosynthesis (*A*), stomatal conductance to CO_2_ (*g*_s_) and internal to ambient [CO_2_] ratio (*C*_i_/*C*_a_) (mean+SE, n = 10) of mountain maple. Seedlings were exposed to two [CO_2_] (392 and 784 µmol mol^−1^), two light levels (100% and 30%) and two soil moisture regimes (high and low) for four months. Means with same letter(s) are not statistically significant (*P*>0.10) from each other. Significant treatment effects are marked as: *P*≤0.01, ***; *P*≤0.05, **; and marginally significant: *P*≤0.10, *.

The interaction between light and soil moisture was significant for *A* and *C*_i_/*C*_a_ and marginally significant for *g*_s_ (Table 1). *A*, *g*_s_ and *C*_i_/*C*_a_ were 28%, 24% and 9% greater respectively, in the high than the low light treatment in seedlings grown at the high soil moisture treatment. In the low soil moisture treatment, however, light treatment had no significant effect on *A*, but high light led to decreased *g*_s_ and *C*_i_/*C*_a_ (by 14%, Fig. 1A, 1B and 1C). In the low light treatment, soil moisture did not have significant effect on *A*, *g*_s_ or *C*_i_/*C*_a_ (Figs. 1A, 1B and 1C). In the high light treatment, however, *A*, *g*_s_ and *C*_i_/*C*_a_ decreased by 29%, 43% and 17%, respectively, in the low than the high soil moisture treatment (Figs. 1A, 1B, and 1C). Furthermore, the elevated [CO_2_] significantly (Table 1) increased *A* by 72% compared with the ambient [CO_2_] but its interaction with light or soil moisture was not significant.

**Table 1:** The *p*-values of ANOVA on the effects of CO_2_ (C), soil moisture (M) and light (L) on net photosynthesis (*A*), stomatal conductance to CO_2_ (*g*_s_), water-use efficiency (IWUE), *C*_i_/*C*_a_ ratio, maximum rate of carboxylation (*V*_cmax_), photosynthetic electron transport rate (*J*), TPU, light compensation point (LCP), apparent quantum efficiency (AQE), dark respiration rates (*R*_d_) and shoot water potential (Ψ_x_) of mountain maple seedlings.

Source of variation	CO_2_	L	CO_2_*L	M	CO_2_*M	L*M	CO_2_*L*M
*A*	**0.0208**	0.1220	0.1630	**0.0015**	0.4976	**0.0049**	0.3142
*g*_s_	0.1585	0.8028	0.8833	**0.0025**	0.2629	**0.0985**	0.7501
IWUE	**0.0327**	**≤0.0001**	**≤0.0001**	0.4910	0.1338	0.4724	0.7446
*C*_i_/*C*_a_	**0.0378**	0.4321	0.5166	0.1210	**0.0039**	**0.0029**	0.4671
*V*_cmax_	0.8930	0.3022	**0.0688**	0.3443	0.1489	0.3211	0.3606
*J*	0.2986	0.5772	**0.0192**	**0.0231**	0.1926	0.3105	0.482
TPU	0.4220	**0.0150**	0.5392	0.6351	**0.0897**	**0.0478**	0.5511
LCP	0.6176	**≤0.0001**	0.6522	0.8859	0.1461	0.9123	**0.0962**
AQE	**0.0389**	0.9785	0.5278	0.5634	0.3213	0.5725	0.3548
*R*_d_	0.1332	**0.0567**	0.5171	0.3160	0.3386	**0.0931**	0.1079
Ψ_x_	**0.0064**	**≤0.0001**	0.8820	**≤0.0001**	**0.0056**	0.6838	0.5897

The seedlings were grown under ambient (392 µmol mol^−1^) or elevated [CO_2_] (784 µmol mol^−1^), 100% or 30% light level. They were exposed to well-watered or water –stressed treatments in 100% and 30% light environments under each [CO_2_]. Measurements were taken after one growing season. Significant effect (*p*≤0.05) and marginally significant effect (*p*≤0.10) are highlighted in bold.

**Figure 2:**
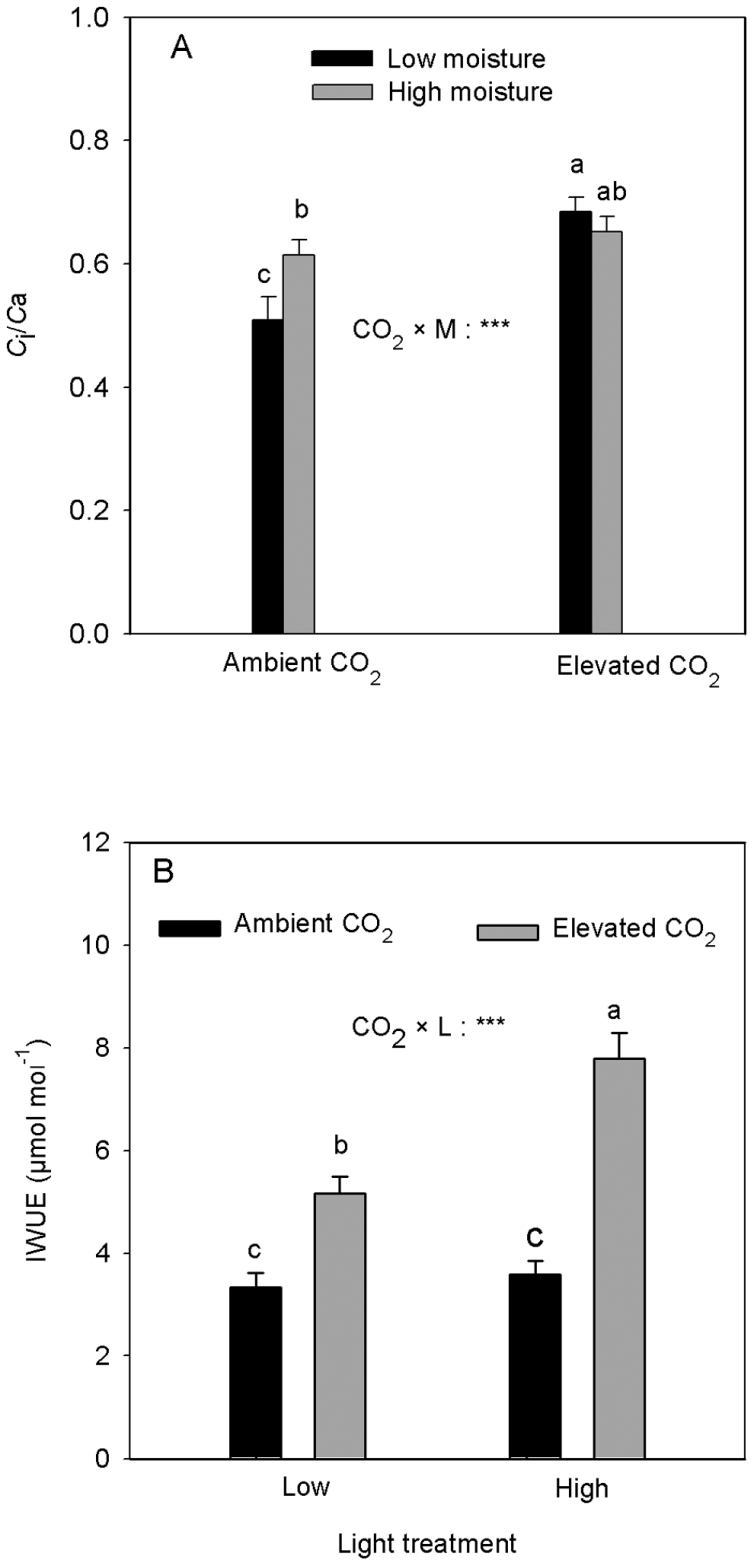
Effects of [CO_2_], L and M on *C*_i_/*C*_a_ ratio and instantaneous water-use efficiency (IWUE) (mean+SE, n = 10) of *Acer spicatum*. The letters on the bars in Fig. 2A represent CO_2_×M interaction and those in Fig. 2B represent CO_2_×L interaction. Refer to Figure 1 for other explanations.

The interaction between CO_2_ and soil moisture had a significant effect on *C*_i_/*C*_a_ (Table 1). *C*_i_/*C*_a_ was significantly lower at the low compared with the high soil moisture under ambient [CO_2_]. However, *C*_i_/*C*_a_ did not differ between soil moisture treatments under the elevated [CO_2_] (Fig. 2A). The elevated [CO_2_] in the low soil moisture treatment significantly increased *C*_i_/*C*_a_ compared with the ambient [CO_2_] (Fig. 2A).

The interaction between CO_2_ and light had a significant effect on IWUE (Table 1). The high light under the elevated [CO_2_] increased IWUE by 51% compared with the low light treatment. However, high light under the ambient [CO_2_] treatment had no significant effect on IWUE (Fig. 2B). The elevated [CO_2_] increased IWUE at both light treatments; the magnitude of increase was however, higher in the high compared with the low light treatment (117% vs. 55%, Fig. 2B).

**Figure 3:**
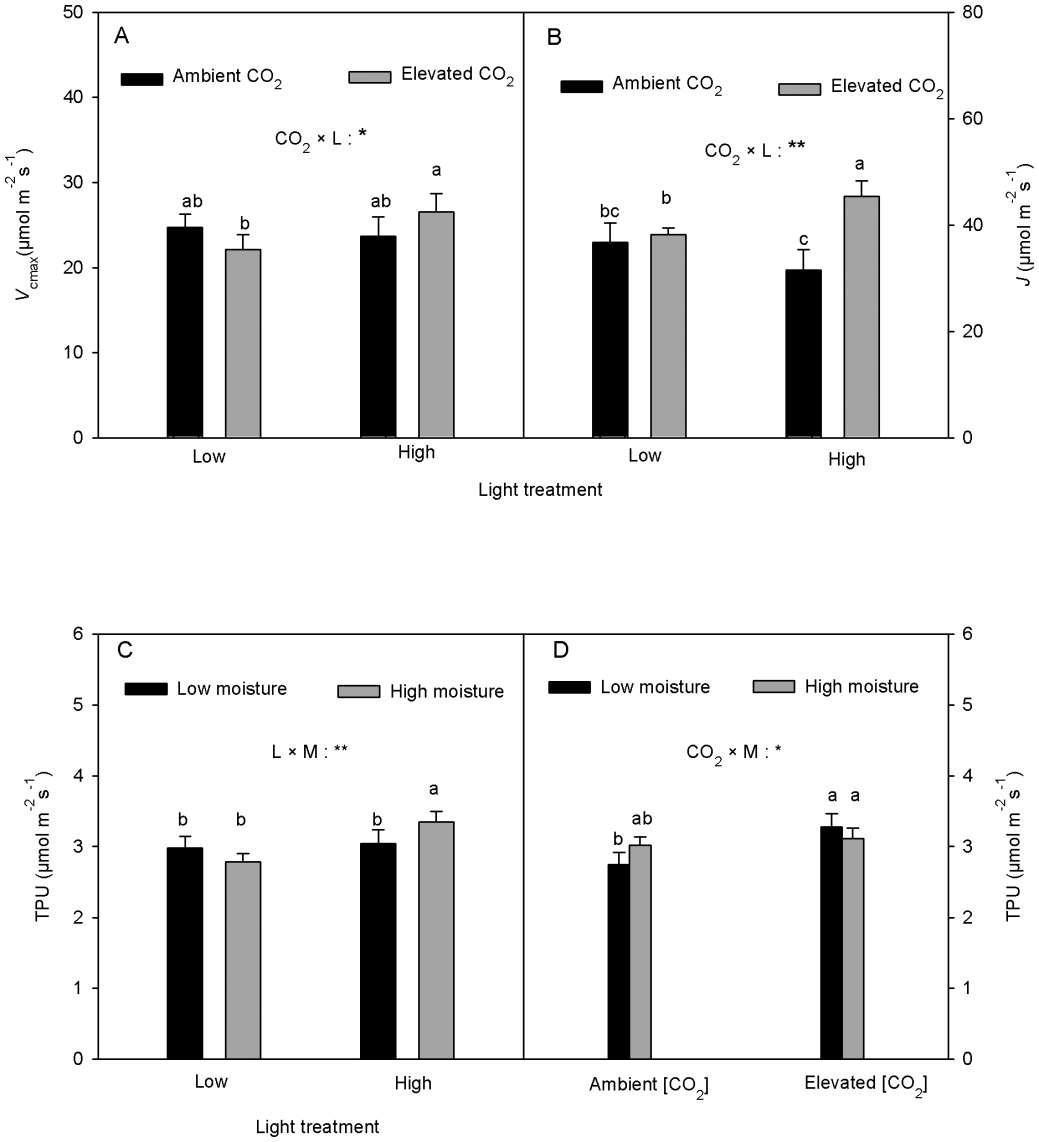
Effects of [CO_2_], and L and M on the maximum rate of carboxylation (*V*_cmax_), photosynthetic electron transport rate (*J*) and triose phosphate utilization (TPU) (mean+SE, n = 10) of *Acer spicatum*. The letters in Figures 3A, 3B and 3C represent CO_2_×L or CO_2_×M interactions. In Fig. 3D, the letters represent L×M interaction. Refer to Figure 1 for other explanations.

The interactive effect of CO_2_ and light on *V*_cmax_ was marginally significant (*p* = 0.0688). The interaction of CO_2_ and light, however, had a significant effect on *J* (Table 1). The high light treatment in the elevated [CO_2_] resulted in higher *V*_cmax_ (20%) and *J* (19%) compared with the low light treatment. However, under the ambient [CO_2_] no significant light effects on *V*_cmax_ or *J* were found (Figs. 3A and 3B). There was no significant [CO_2_] affect on *V*_cmax_ in either light treatments or on *J* in the low light treatment (Figs. 3A and 3B). The elevated [CO_2_] in the high light treatment significantly increased *J* by 44% compared with the ambient [CO_2_] (Fig. 3B).

TPU was significantly affected by the light and soil moisture interaction and marginally significantly affected by the interaction between CO_2_ and soil moisture (Table 1). The high light treatment significantly increased TPU (20% higher than in the low light ) at the high soil moisture but had no significant effect at the low soil moisture (Fig. 3C). Furthermore, the low soil moisture decreased TPU (9% lower than the high soil moisture) under the high light but had no significant effect at the low light treatment (Fig. 3C). Soil moisture did not significantly affect TPU under either [CO_2_] (Fig. 3D). The elevated [CO_2_] significantly increased TPU by 19% than in the ambient [CO_2_] at the low soil moisture but had no significant effect at the high soil moisture (Fig. 3D).

**Figure 4:**
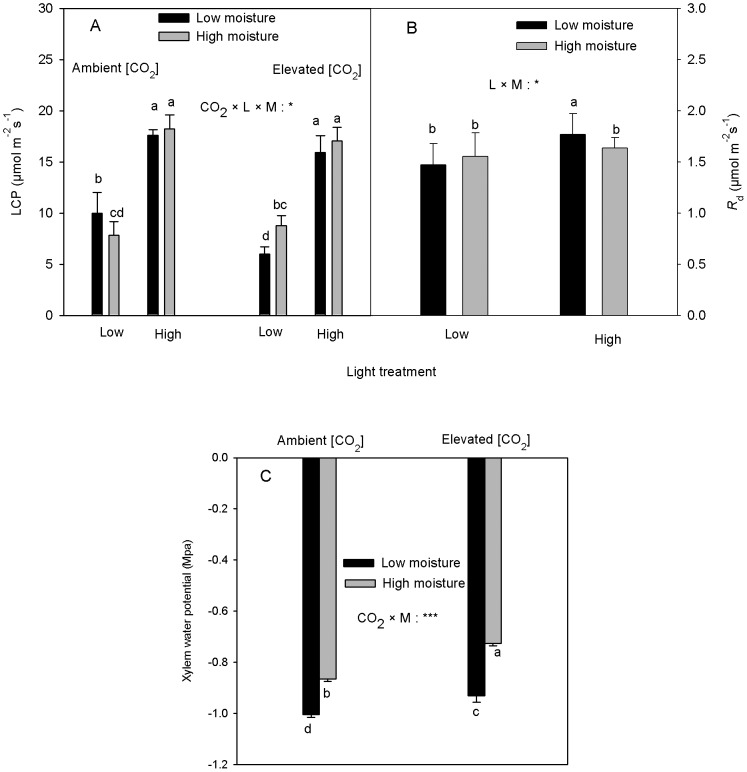
Effects of [CO_2_], L and M on photosynthetic light compensation point (LCP), dark respiration rate (*R*_d_) and midday xylem water potential (mean+SE, n = 10) of *Acer spicatum*. The lower case letters in Figure 4A represent CO_2_×L×M interactions. In Fig. 4B, the letters represent L×M interaction and the letters in Fig. 4C represent CO_2_×M interactions. Refer to Figure 1 for other explanations.

There was a marginal significant interactive effect among CO_2_, light and soil moisture on LCP (Table 1). LCP was higher in the high than in the low light treatment in all the CO_2_ and soil moisture combinations (Fig. 4A). The low soil moisture increased LCP under the ambient [CO_2_] and low light treatment while it showed an opposite effect on LCP under the elevated [CO_2_] and low light treatment (Fig. 4A). No significant soil moisture effect on LCP was found at the high light under either [CO_2_]. The elevated [CO_2_] significantly reduced LCP only in the low soil moisture and low light combination but had no significant effect on LCP at the other treatment combinations (Fig. 4A).

The elevated [CO_2_] significantly increased the AQE of photosynthesis (24% higher than under ambient [CO_2_]) but no other significant effects on AQE were found (Table 1). The interaction between light and soil moisture had a marginally significant effect on *R*_d_ (Table 1). The high light treatment increased *R*_d_ at the low soil moisture but not in the high soil moisture treatment (Fig. 4B). The low soil moisture significantly increased Rd at high light but had significantly effect on *R*_d_ at the low light condition (Fig. 4B).

The interaction between [CO_2_] and soil moisture significantly affected shoot midday Ψ_x_ (Table 1). Ψ_x_ was significantly more negative at the low soil moisture than at the high soil moisture and the magnitude of the difference was greater under elevated than ambient [CO_2_] (Fig. 4C). Furthermore, the elevated [CO_2_] significantly increased Ψ_x_ (less negative) at both soil moisture treatments, but the magnitude was greater in the high (19%) than low (8%) soil moisture (Fig. 4C). The high light treatment significantly decreased Ψ_x_ (−0.93 at high light vs. −0.83 at low light, Table 1).

## Discussion

This study shows that the net photosynthesis (*A*) of mountain maple seedling would be less responsive to canopy gaps when soil moisture is low and that seedlings would be more sensitive to moisture stress when grown in canopy gaps than when grown in shade of a forest canopy. It is a little surprising that *A* in the low soil moisture did not show any significant response to the light treatment. However, the results that the low soil moisture conditions reduced *A* only in the seedlings grown in the high light supports our hypothesis that the low soil moisture would limit *A* to a greater extent in high than low light treatment. The results are in agreement with other studies that reported reduced *A* response to high light condition when soil moisture was low [51], [52]. It was expected decreased *g*_s_ or leaf area in the high light under low soil moisture would have negative effects on *A*. Although *g*_s_ showed a trend towards a decrease, there was no significant difference between the low and high light under the low soil moisture. Similarly, the high light treatment did not decrease leaf area production when seedlings were exposed to low soil moisture (Danyagri and Dang *unpublished*). The results show that a reduction in soil moisture supply may limit the net photosynthetic response of mountain maple seedlings to high light conditions associated with the occurrence of canopy gaps. In addition, multiple resource limitations may act to affect mountain maple physiological performances in high light conditions [47].

Both stomatal and non-stomatal limitation to *A* in response to high light under the low soil moisture was found in this study, but one of them appeared to be the primary limiting factor. The relative limitations by stomatal and non-stomatal factors to *A* are reported to vary with species and treatment [38], [53], [54]. The fact that *C*_i_/*C*_a_ was significantly lower in the high than low light treatment under the low soil moisture treatment suggests that stomatal factors were the primary limitation to *A* response to light. Water stress has been reported to decrease the amounts of ATP and ribulose bisphosphate in the leaf, leading to reduced *A* in other plant species [54]. However, the low soil moisture did not reduce the rate of triose phosphate utilization in seedlings grown at the high light treatment in this study, which further suggests that stomata were probably the primary factors limiting the *A* response to the high light treatment in this species. The results may indicate that the biochemical apparatus of *A* in mountain maple seedlings was not impaired or less impacted than the stomata by the high light in low soil moisture treatment.

We observed a significant synergistic effect of high light and elevated [CO_2_] on instantaneous water use efficiency. While the elevated [CO_2_] significantly increased IWUE in both light treatments, the high light treatment increased IWUE only under the elevated [CO_2_]. An increase in either photosynthesis or a decrease in transpiration rate or a combination of both by elevated [CO_2_] have been reported to increase IWUE [19], [25], [55]. In this study, the interaction between [CO_2_] and light did not significantly affect *A* and *g*_s_ (log transpiration (*E*) dataset is provided as supplementary data, Table S1). It appears that other factors other than leaf gas exchange may have been responsible for the significant increase in IWUE under elevated [CO_2_] and high light treatment. Norby and O’Neill [56] reported that morphological modifications of *Liriodendron tulipifera* L. seedlings under elevated [CO_2_] had a larger effect on WUE than did gas exchange. Hence, it is possible that morphological adjustments could be the contributing factor in the observed IWUE response to elevated [CO_2_] in high light in this study.

Another interesting observation in this study was that seedlings grown in the high light treatment showed increases in *V*_cmax_ and *J* under elevated [CO_2_]. *V*_cmax_ and *J* generally decrease in plants grown under elevated [CO_2_] (i.e. down-regulation), although there are some exceptions [16], [17]. In an open-air CO_2_ enrichment study, there was an increase in photosynthetic capacity and a lack of down-regulation at the upper-canopy of young deciduous forest due to the close proximity to rapidly growing shoot (sink for photosynthesis) [57]. In this study, new leaves were repeatedly initiated throughout the experiment, and the leaves used for the gas exchange measurements were all from the top canopy position. Our results suggest that mountain maple seedlings exposed to high light may be able to maintain high photosynthetic capacity under elevated [CO_2_].

It is interesting that low soil moisture did not significantly affect the light compensation point for photosynthesis under the high light and elevated [CO_2_] but reduced it under the ambient [CO_2_]. Although the LCP generally increase in response to the high light treatment, particularly under elevated [CO_2_], the greatest increase occurred in the elevated [CO_2_] and low soil moisture treatment combination. We expected that increased respiration under low soil moisture would result in a higher LCP. The low soil moisture treatment increased *R*_d_ at the high light, but there was no corresponding increase in LCP. Similarly, there was no significant interaction among [CO_2_], light and soil moisture on the apparent quantum yield of photosynthesis. However, since LCP alone does not necessarily determine plant ability to maintain positive carbon balance [58], [59], it is unclear whether the growth potential of mountain maple may be limited by low soil moisture conditions when seedlings are exposed to high light in canopy gaps under higher [CO_2_] in the future.

The results demonstrate that further increases in atmospheric [CO_2_] may enhance the drought tolerance of mountain maple seedlings. The mid-day xylem water potential was significantly higher (i.e. less negative) under the elevated than the ambient [CO_2_]. Increased osmotic adjustment may be the mechanism responsible for the increased drought tolerance under elevated [CO_2_]. Tschaplinski *et al*. [60] found that elevated [CO_2_] increased osmotic potential at turgor loss point in loblolly pine seedlings grown at low and high soil moisture by 17.42% and 17.02%, respectively. Furthermore, they found that the elevated [CO_2_] also increased biomass allocation to root, which could potentially mitigate the drought effect and enhance continued growth. In this study, the elevated [CO_2_] increased biomass allocation to root, and the magnitude of increase was greater under the low than the high soil moisture (Danyagri and Dang *unpublished*). These results are in agreement with the findings of other studies that found increased biomass allocation to root as a drought tolerant mechanism under low soil moisture and elevated [CO_2_] [61].

In conclusion, mountain maple seedlings generally responded positively to the high light and elevated [CO_2_] in this study. The positive effects of high light and elevated [CO_2_] on *A* and IWUE were substantial, indicating that mountain maple seedlings in canopy gaps may benefit from further increases in atmospheric [CO_2_]. Although many factors act to influence growth, morphological adjustment and higher photosynthetic capacity may be the main contributing factors. The results further indicate that elevated [CO_2_] would ameliorate the negative effect of low soil moisture on mountain maple seedlings growth.

## Supporting Information

Table S1 **LI-COR 6400 logged transpiration dataset of mountain maple seedlings grown for one season.** The treatments were: ambient [CO_2_], high light and high soil moisture (AHLHM), ambient [CO_2_], low light, and high soil moisture (ALLHM), ambient [CO_2_], low light and low soil moisture (ALLLM), or elevated [CO_2_], high light and high soil moisture (EHLHM), elevated [CO_2_], low light and high soil moisture (ELLHM), and elevated [CO_2_], low light and low soil moisture (ELLLM). There were two replications (R) for each [CO_2_] treatment. Gas exchange measurements were carried out on five seedlings in each treatment combination at seven CO_2_ concentrations.
(XLSX)
